# Interlukin-22 improves ovarian function in polycystic ovary syndrome independent of metabolic regulation: a mouse-based experimental study

**DOI:** 10.1186/s13048-024-01428-x

**Published:** 2024-05-11

**Authors:** Weixuan Chen, Baoying Liao, Chuyu Yun, Min Zhao, Yanli Pang

**Affiliations:** 1 State Key Laboratory of Female Fertility Promotion, Center for Reproductive Medicine, Department of Obstetrics and Gynaecology, Peking University Third Hospital, Beijing, China; 2 National Clinical Research Center for Obstetrics and Gynaecology, (Peking University Third Hospital), Beijing, China; 3 Key Laboratory of Assisted Reproduction (Peking University), Ministry of Education, Beijing, China; 4 Beijing Key Laboratory of Reproductive Endocrinology and Assisted Reproductive Technology, Beijing, China

## Abstract

**Background:**

Polycystic ovary syndrome (PCOS) is a reproductive endocrine disorder with multiple metabolic abnormalities. Most PCOS patients have concomitant metabolic syndromes such as insulin resistance and obesity, which often lead to the development of type II diabetes and cardiovascular disease with serious consequences. Current treatment of PCOS with symptomatic treatments such as hormone replacement, which has many side effects. Research on its origin and pathogenesis is urgently needed. Although improving the metabolic status of the body can alleviate reproductive function in some patients, there is still a subset of patients with metabolically normal PCOS that lacks therapeutic tools to address ovarian etiology.

**Methods:**

The effect of IL-22 on PCOS ovarian function was verified in a non-metabolic PCOS mouse model induced by dehydroepiandrosterone (DHEA) and rosiglitazone, as well as granulosa cell -specific STAT3 knockout (*Fshr*^*cre+*^*Stat3*^*f/f*^) mice (10 groups totally and *n* = 5 per group). Mice were maintained under controlled temperature and lighting conditions with free access to food and water in a specific pathogen-free (SPF) facility. Secondary follicles separated from *Fshr*^*cre+*^*Stat3*^*f/f*^ mice were cultured in vitro with DHEA to mimic the hyperandrogenic environment in PCOS ovaries (4 groups and *n* = 7 per group) and then were treated with IL-22 to investigate the specific role of IL-22 on ovarian function.

**Results:**

We developed a non-metabolic mice model with rosiglitazone superimposed on DHEA. This model has normal metabolic function as evidenced by normal glucose tolerance without insulin resistance and PCOS-like ovarian function as evidenced by irregular estrous cycle, polycystic ovarian morphology (PCOM), abnormalities in sex hormone level. Supplementation with IL-22 improved these ovarian functions in non-metabolic PCOS mice. Application of DHEA in an in vitro follicular culture system to simulate PCOS follicular developmental block and ovulation impairment. Follicles from *Fshr*^*cre+*^*Stat3*^*f/f*^ did not show improvement in POCS follicle development with the addition of IL-22. In DHEA-induced PCOS mice, selective ablation of STAT3 in granulosa cells significantly reversed the ameliorative effect of IL-22 on ovarian function.

**Conclusion:**

IL-22 can improve non-metabolic PCOS mice ovarian function. Granulosa cells deficient in STAT3 reverses the role of IL-22 in alleviating ovary dysfunction in non-metabolic PCOS mice.

**Supplementary Information:**

The online version contains supplementary material available at 10.1186/s13048-024-01428-x.

## Introduction

PCOS is a complex reproductive endocrine disease, and many studies have shown that PCOS patients are accompanied by metabolic disorders, and as the incidence of the disease gradually increases, a large proportion of PCOS patients have only ovarian dysfunction, Side effects of symptomatic treatments such as hormone replacement are high in these patients. However, there are no relative targeting of etiology for treatment [1]. Using biochemical features and genotype data of 893 PCOS patients, Dapas et al. classified two distinct reproductive and metabolic subtypes of women with PCOS [2]. The metabolic PCOS subtype (37-39%) is characterized by a high BMI and insulin resistance with relatively normal LH and sex hormone binding globulin (SHBG) levels [2–4]. Symptom-oriented treatment, such as lifestyle modification or insulin sensitizers, can effectively improve the clinical pregnancy outcomes of PCOS patients with metabolic disorders [5, 6]. Moreover, 21-23% of PCOS patients are defined as the reproductive subtype, characterized by impaired reproductive function, including abnormally increased LH and androgen levels, with normal BMI and metabolic status [2]. For these patients, the consequently enhanced LH/FSH ratio and excessive androgen may impair follicular development [1], leading to the developmental arrest and accumulation of small antral follicles with a diameter of 2–8 mm in the ovary, causing polycystic changes.

IL-22 is a member of the IL-10 family and has been widely reported to alleviate metabolic disorders [7]. IL-22 has been shown to restore insulin resistance in obesity by inhibiting pancreatic beta cell apoptosis and enhance insulin sensitivity by promoting peripheral white fat browning [8]. In addition, serum levels of IL-22 have been associated to some extent with obesity, although research findings vary [7, 9, 10]. Thus research into IL-22 function has triggered a new surge in endocrine metabolic disorders [11, 12]. Regardless, due to the extensive chronic low-grade inflammatory response in PCOS, IL-22 has been reported by many studies to alleviate PCOS by improving systemic inflammation levels as well as insulin resistance [13, 14]. Our research group has previously found that administration of IL-22 to different PCOS mice alleviated insulin resistance and thus improved ovarian function [10]; However, it remains unclear whether IL-22 has a directly effect on the ovary and whether it has a positive effect on the treatment of patients with non-metabolic PCOS.

Here, we first established a non-metabolic PCOS mouse model to exclude the effect of metabolic factors and further clarified that IL-22 relies on STAT3 to ameliorate follicle development and ovulation of non-metabolic PCOS mice by constructing granulosa cell-specific STAT3 knockout (*Fshr*^*cre+*^*Stat3*^*f/f*^) mice.

## Materials and methods

### Animals and study design

Wildtype C57BL/6J female mice were purchased from Department of Experimental Animal Science, Peking University Health Science Center. *Stat3*^*fl/fl*^ mice and *Fshr-Cre* mice were kindly provided by Hua Yu from (Beckman Research Institute of City of Hope, CA) and Youqiang Su (Shandong University, Qingdao, China), respectively. Since the FSH receptor promoter is only expressed in granulosa cells, we generated granulosa cell-specific STAT3 knockout mice to better explore the specific mechanisms by which IL-22 affects granulosa cells by crossing *Stat3*^*fl/fl*^ mice with *Fshr-Cre* mice [15]. The mice were maintained under controlled temperature and lighting conditions (12-h light:12-h darkness cycle) with free access to food and water in a specific pathogen-free (SPF) facility. Three-week-old wildtype C57BL/6J female mice, which weighed 10–15 g, were randomly divided into 6 groups: Control, DHEA, DHEA + rosiglitazone for establishing a non-metabolic PCOS mouse model; Control, DHEA + rosiglitazone, DHEA + rosiglitazone + IL-22 for clarifying the role of IL-22 in a non-metabolic PCOS mouse model; Three-week-old *Fshr*^*cre−*^*Stat3*^*f/f*^*and Fshr*^*cre+*^*Stat3*^*f/f*^ female mice, which weighed 10–15 g, were randomly divided into 4 groups: *Fshr*^*cre−*^*Stat3*^*f/f*^*+*DHEA, *Fshr*^*cre−*^*Stat3*^*f/f*^*+*DHEA + IL-22, *Fshr*^*cre+*^*Stat3*^*f/f*^*+*DHEA, *Fshr*^*cre+*^*Stat3*^*f/f*^*+*DHEA + IL-22 for demonstrating that IL-22 depends on STAT3 to directly improve ovarian function. Mice in the DHEA group were injected daily with DHEA (Sigma, D4000) (6 mg/100 g body weight, dissolved in 0.1 mL of sesame oil) subcutaneously for 21 days. After 3 weeks of treatment, these mice received another 3 weeks of treatment with rosiglitazone (3 mg/kg/d, Aladdin, R129756) by oral gavage for the non-metabolic PCOS mouse model (DHEA + rosiglitazone group). On this basis, we further investigated the possible effect of IL-22 on PCOS ovaries. IL-22 (100 µg/kg/d, R&D) or PBS administration was initiated at the same time as rosiglitazone gavage to mice. Before the mice were anaesthetized with ether and killed by cervical dislocation, the animals from each group were subjected to a glucose tolerance test (GTT) and insulin tolerance test (ITT). The mice were anaesthetized with ether and killed by cervical dislocation after a 12-h fast. Serum was collected to measure insulin and sex hormone levels. The ovaries were processed for haematoxylin and eosin staining. The serum was quickly frozen and stored at − 80 °C.

### Vaginal smears and estrous cycle determination

Vaginal smears were performed for 10 consecutive days (at least 2 cycles) from the 10th to the 19th day after the first day of treatment. After Shorr staining, the specific stage of the estrous cycle was identified by analysing vaginal cytology under a microscope.

### Glucose tolerance test (GTT) and insulin tolerance test (ITT)

Mice were fasted for 12 h before the GTT experiment and for 4 h before the ITT experiment. Glucose levels were measured by tail vein blood sampling using a blood glucose Accu-Chek Performa (Roche Diagnostics). After measurement of fasting glucose levels, glucose (2 g/kg body weight) or insulin (1 IU/kg body weight) was injected intraperitoneally (timepoint 0 min), and glucose levels were measured from tail sampling at the 15-, 30-, 60- and 90-min time points according to a previous study [10].

### Measurement of serum hormones levels

The blood samples were centrifuged at 4 °C for 10 min at 3000 rpm, and the serum was separated and stored at − 80 °C for subsequent serum determinations. As described previously [13], the levels of testosterone (S10940093, range: 0.1–20 ng/mL, sensitivity: 0.02 ng/mL), luteinizing hormone (LH, S10950161, range: 5–200 mIU/mL, sensitivity: ≤1.0 mIU/mL), estradiol (20,172,400,011, range: 5–4000 pg/mL, sensitivity: ≤2 pg/mL), follicle-stimulating hormone (FSH, S10950154, range: 2.5–100 mIU/mL, sensitivity: <1.0 mIU/mL).

### Ovarian histology

Ovaries were collected and fixed in 4% paraformaldehyde (PFA), gradient dehydrated with ethanol and embedded in paraffin. Paraffin-embedded ovaries were serially sectioned into 5-µm sections (CM1850; Leica) and then processed for haematoxylin and eosin staining. The numbers of corpora luteum and cystic follicles were counted under a light microscope (NIS-Elements 3.2, Nikon Eclipse 80i; Nikon).

### Mouse granulosa cell collection

Eight- to ten-week-old female mice were intraperitoneally injected with 5–10 IU pregnant mare serum gonadotropin (PMSG, Shusheng, Ningbo, China) 46–48 h before granulosa cell collection. Forty-eight hours after PMSG injection, the ovaries were separated, and the follicles were punctured with a syringe to discharge the cell mass of granulosa cells. Granulosa cells were collected with a mouth pipette, washed with DPBS, cultured in DMEM-F12 supplemented with 10% FBS and 1% PS for 12 h, and subsequently collected for RNA extraction.

### *In vitro* culture and maturation of mouse ovarian follicles

Intact secondary follicles with a diameter of 180–200 μm were mechanically separated from 18- to 21-day-old female mouse ovaries. Separated follicles were incubated in αMEM (32,571,036, Sigma‒Aldrich) supplemented with 1% FBS for 1 h, capsulated with 0.5% alginate (Sigma‒Aldrich) and cultured in 96-well plates with αMEM supplemented with 3 mg/ml BSA (B2064, Sigma‒Aldrich), 1 mg/ml bovine fetuin (F2379, Sigma‒Aldrich), 10 mIU/ml recombinant follicular stimulating hormone, 5 µg/ml insulin, 5 µg/ml transferrin, and 5 µg/ml selenium (I3146, Sigma‒Aldrich) for 6 days. DHEA (0.01 mM; HY-14,650; Med Chem Express), IL-22 (100 ng/ml) was added to the growth medium. Half of the growth medium was changed every 2 days, and follicles were imaged by fluorescence microscopy at each media change.

For in vitro maturation, follicles were released from alginate beads and incubated in αMEM supplemented with 10% FBS, 1% PS, 1.5 IU/ml human chorionic gonadotropin (hCG) and 10 ng/ml epidermal growth factor (EGF, PHG0311, Gibco) for 18 h. After 18 h of hCG treatment, follicles, ovulated COCs and oocytes were imaged. Oocytes with first polar body extrusion were classified as mature oocytes. The ovulation of 7 follicles was counted to calculate the ovulation rate and maturation rate each time. Every 3 follicles were collected for RNA extraction using a RNeasy Mini Kit (74,104, QIAGEN).

### RNA extraction and RNA sequencing (RNA-seq) analysis

Total RNA was extracted from collected mouse granulosa cells with TRIzol reagent (15,596,018; Life Technologies) according to the manufacturer’s protocol. Total RNA from three mice was mixed as one sample for RNA-seq analysis. As described previously [16], the clustering of the index-coded samples was performed on a cBot Cluster Generation System using the TruSeq PE Cluster Kit v3-cBot-HS (Illumina) according to the manufacturer’s instructions. After cluster generation, the library preparations were sequenced on an Illumina NovaSeq platform, and raw data (raw reads) in fastq format were first processed through in-house Perl scripts. At the same time, the Q20, Q30 and GC contents of the clean data were calculated. All downstream analyses were based on clean data with high quality. The index of the reference genome was built using Hisat2 v2.0.5 and paired-end clean reads were aligned to the reference genome using Hisat2 v2.0.5. Bioinformatic analysis was performed using the OmicStudio tools at https://www.omicstudio.cn/tool.

### cDNA synthesis and quantitative real-time PCR analysis

One hundred nanograms of RNA was reverse transcribed to cDNA using the RevertAid First cDNA Synthesis Kit (K1622; Thermo Scientific) according to the manufacturer’s protocols. The primers used for real-time qPCR are listed in Table S1. Real-time qPCR was performed in an ABI 7500 real-time PCR system (Applied Biosystems) using SYBR Green PCR Master Mix (Invitrogen). The relative expression level of genes was normalized to that of 18 S RNA.

### Statistical analysis

All statistical analyses were carried out using SPSS version 24 and visualised with GraphPad Prism version 8.0 (GraphPad Software). For parametric tests, two-tailed Student’s t test was used to evaluate the statistical significance between two groups, and one-way ANOVA followed by Tukey’s post hoc test was used for three or more groups. For nonparametric tests, the Kruskal–Wallis test was used to analyse the differences among three or more experimental groups, followed by Dunn’s post hoc analysis. Data are shown as the means ± SEMs or as the medians with interquartile ranges. *P* < 0.05 was considered statistically significant.

## Results

### Rosiglitazone treatment contributed to the establishment of a non-metabolic PCOS mouse model

In previous studies, although many types of PCOS animal models have been used, due to the unidentified aetiology and complex pathogenesis of PCOS, none of them could simulate the phenotypes of PCOS patients without metabolic disorders [17]. In addition, IL-22 plays an important role in alleviating the metabolic syndrome by improving insulin resistance [11]. Specifically, IL-22 restores insulin secretion in obese mice by protecting pancreatic beta cells from oxidative and endoplasmic reticulum stress [7, 8]. Whether IL-22 can play a role other than improving metabolism in PCOS remains unknown. To clarify the role of IL-22 in non-metabolic PCOS patients, we needed to establish a mouse model to simulate this condition. Here we first applied rosiglitazone, a potent thiazolidinedione and insulin sensitizer, to establish a non-metabolic PCOS mouse model based on a DHEA-induced PCOS mouse model.

**Fig. 1:**
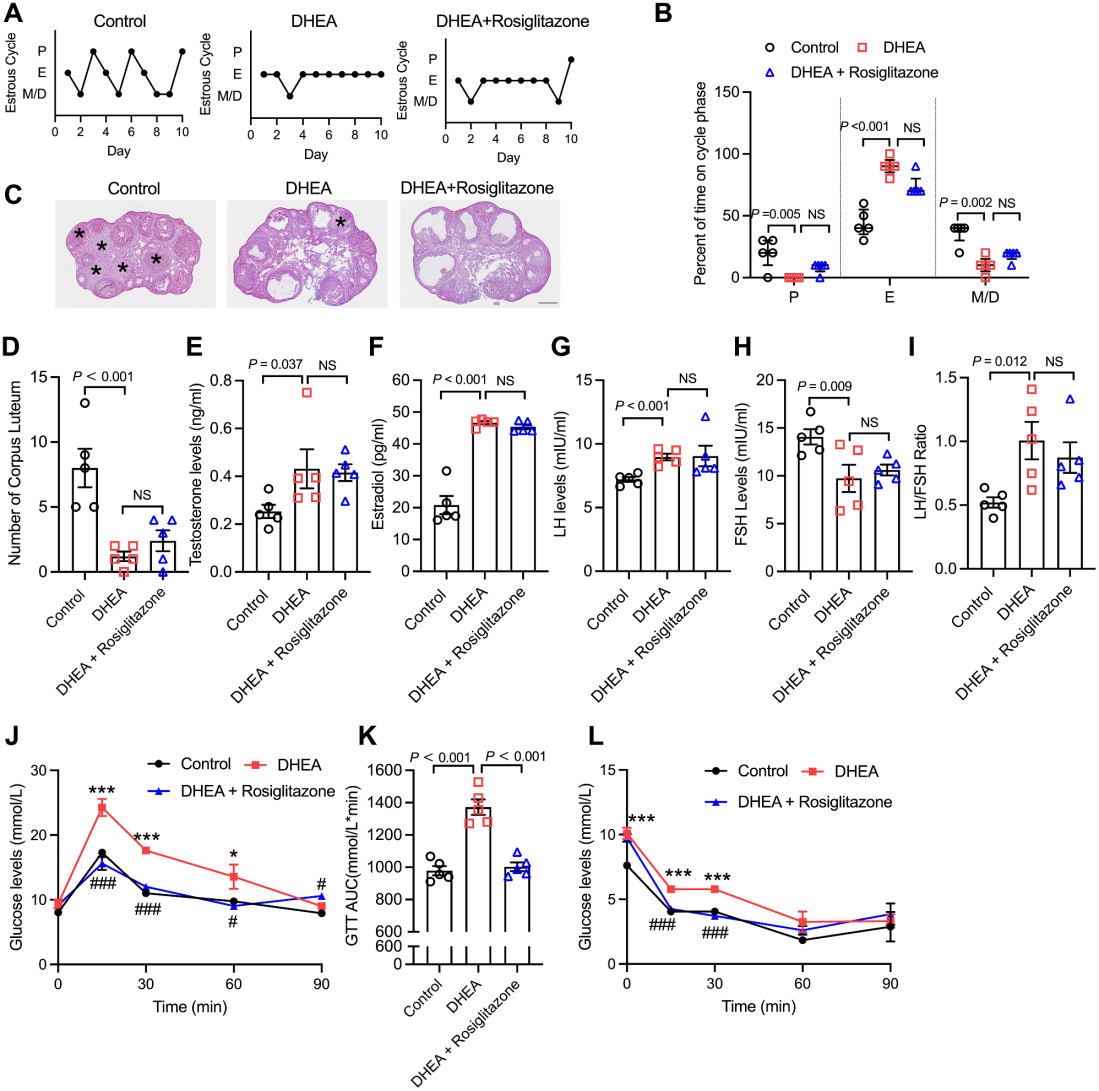
Rosiglitazone treatment contributed to the establishment of a non-metabolic PCOS mouse model. (**A**) Representative estrous cycles of mice in the control, DHEA and DHEA + rosiglitazone groups. (**B**) Quantitative analysis of estrous cycles (data are presented as the medians with interquartile ranges, *n* = 5 mice per group). (**C**) Haematoxylin and eosin staining of representative ovaries. The corpora luteum are indicated by asterisks. Images are representative of three independent experiments with similar results. Scale bar: 250 μm. (**D**) Quantitative analysis of corpora luteum (*n* = 5 mice per group). (**E**-**H**) Serum levels of testosterone, estradiol, LH and FSH in mice in the control, DHEA and DHEA + rosiglitazone groups (*n* = 5 mice per group). (**I**) The ratio of LH/FSH. (**J** and **K**) GTT and area under the curve of the GTT (*n* = 5 mice per group). (**L**) ITT (*n* = 5 mice per group). The number of biological replicates is indicated as ‘n’. P, proestrus; E, estrous; M/D, metestrus/diestrus. GTT, glucose tolerance test; ITT, insulin tolerance test. Data are presented as the means ± SEMs. For (**J** and **L**) *P < 0.05; ***P < 0.001 versus the control group. #*P* < 0.05; ###*P* < 0.001 versus the DHEA + rosiglitazone group

First, we examined the reproductive phenotype of the PCOS mouse model. DHEA-treated mice showed an apparently irregular estrous cycle and a significantly elevated percentage of estrous time in estrous cycles compared with those of the control group; supplementation with rosiglitazone seemed to have little impact on the estrous cycle (Fig. 1A, B). Ovaries from mice in the control group consisted of growing follicles and corpus luteum, while ovaries were polycystic-like in the DHEA and DHEA + rosiglitazone groups (Fig. 1C), with significantly decreased corpus luteum numbers (Fig. 1D), which indicated the disruption of follicle development and ovulation in these two groups. The results of serum hormone level measurement showed that serum testosterone levels (Testosterone is androgen with high bioactivity which is the primary indicator of hyperandrogenism in PCOS. So we picked testosterone as the androgen representative.) and the LH/FSH ratio were increased and FSH levels were decreased in the DHEA-induced PCOS mouse model, and rosiglitazone treatment decreased the LH/FSH ratio in PCOS mice to some extent but did not reach significance (Fig. 1E-I). Mice in the DHEA group displayed impaired glucose tolerance and insulin resistance according to the results of the GTT and ITT (Fig. 1J, L). The area under the curve (AUC) of the GTT results was increased in DHEA-treated mice (Fig. 1K), while rosiglitazone completely reversed the DHEA-induced metabolic dysfunction in the PCOS mouse model. Overall, with rosiglitazone superimposed on the DHEA model, mice exhibited only abnormal ovarian function and no metabolic abnormality phenotype. It indicates that the non-metabolic PCOS mouse model was successfully constructed.

### IL-22 improved ovarian function in a non-metabolic PCOS mouse model

**Fig. 2:**
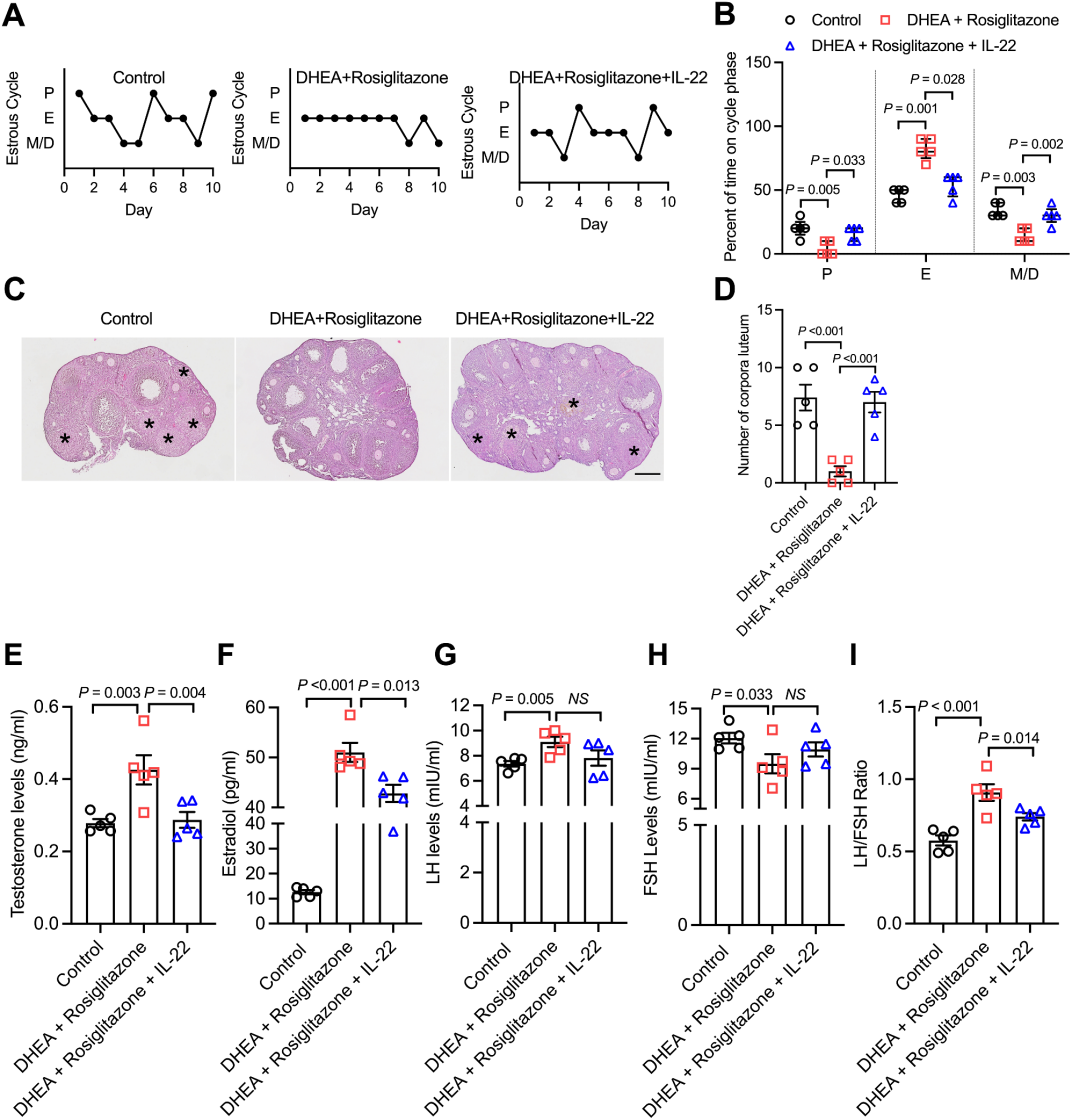
IL-22 improved ovarian function in a non-metabolic PCOS mouse model. (**A**) Representative estrous cycles of mice in the control, DHEA + rosiglitazone and DHEA + rosiglitazone + IL-22 groups. (**B**) Quantitative analysis of estrous cycles (data are presented as the medians with interquartile ranges, *n* = 5 mice per group). (**C**) Haematoxylin and eosin staining of representative ovaries. The corpora luteum are indicated by asterisks. Images are representative of three independent experiments with similar results. Scale bar: 250 μm. (**D**) Quantitative analysis of corpora luteum (*n* = 5 mice per group). (**E**-**H**) Serum levels of testosterone, estradiol, LH and FSH in mice in the control, DHEA and DHEA + rosiglitazone groups (*n* = 5 mice per group). (**I**) The ratio of LH/FSH. The number of biological replicates is indicated as ‘n’. P, proestrus; E, estrous; M/D, metestrus/diestrus. Data are presented as the means ± SEMs

To clarify the role of IL-22 in a non-metabolic PCOS mouse model, we further administered IL-22 to mice in the DHEA + rosiglitazone group and found that the estrous cycle alternated regularly after IL-22 treatment (Fig. 2A). In comparison with the DHEA + rosiglitazone group, mice in the DHEA + rosiglitazone + IL-22 group had a lower percentage of E and a higher percentage of P and M/D (Fig. 2B). The results of ovarian histology revealed that IL-22 significantly alleviated PCOM in non-metabolic PCOS mice and enhanced the corpus luteum numbers (Fig. 2C, D). Moreover, IL-22 was able to lower testosterone levels and enhance estradiol levels to those in the control group (Fig. 2E, F). Regardless of the supplementation of IL-22, the FSH or LH levels were not significantly different; however, IL-22 significantly reduced the LH/FSH ratio (Fig. 2G-I). The above findings indicated that IL-22 noticeably alleviated ovarian disorders, including irregular estrous cycles, polycystic ovaries and aberrant hormone secretion, in non-metabolic PCOS mice in a manner that was relatively independent of improved metabolic function.

### Granulosa cell deletion of STAT3 reverses the ameliorative effect of IL-22 on follicular development and ovulation

**Fig. 3:**
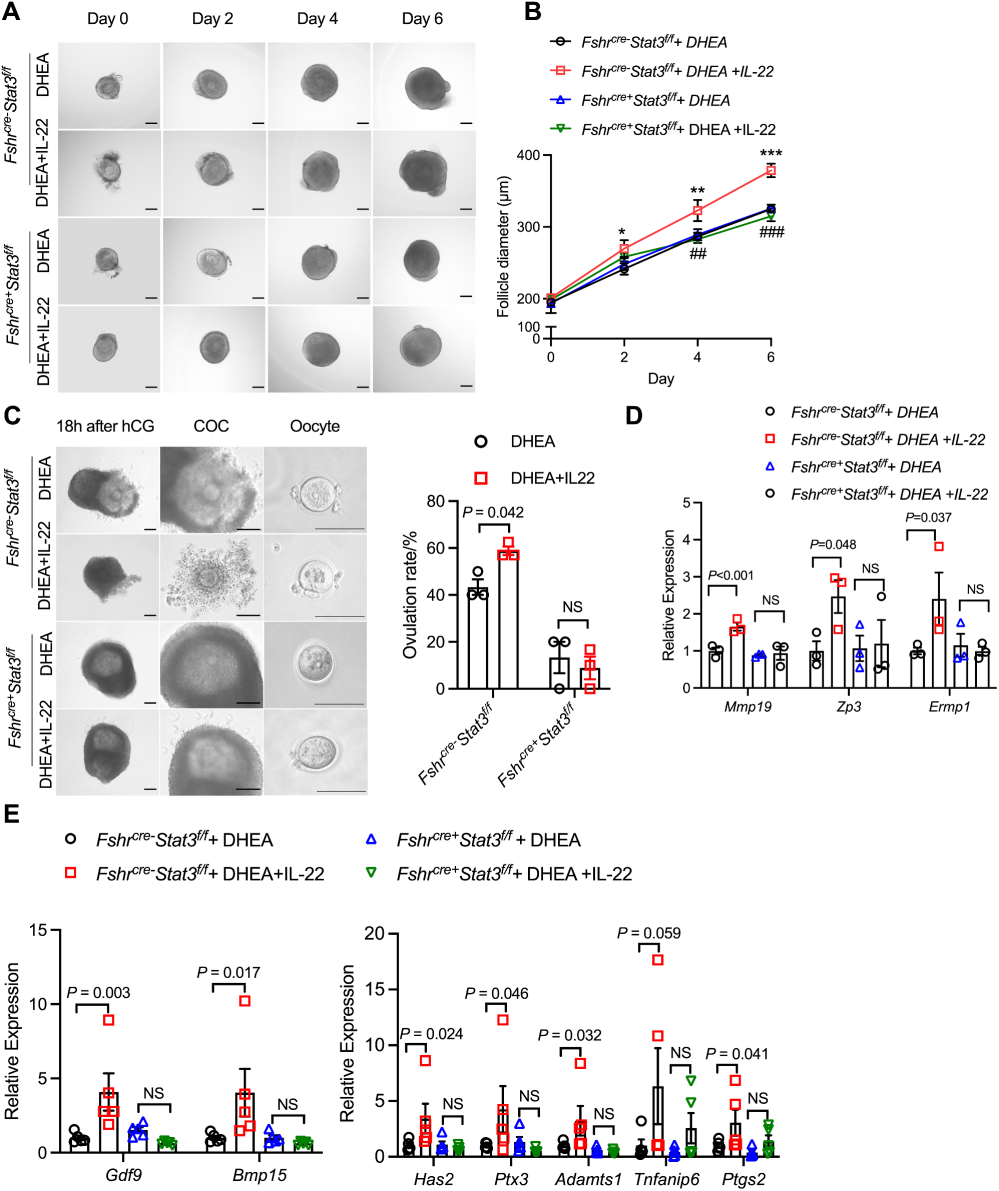
IL-22 depends on STAT3 to improve follicular development and ovulation in PCOS mice. (**A**) Representative micrograph of DHEA- and IL-22-treated follicles separated from *Fshr*^*cre−*^*Stat3*^*f/f*^ mice and *Fshr*^*cre+*^*Stat3*^*f/f*^ mice cultured in vitro. Scale bar: 100 μm. (**B**) Diameters of follicles (*n* = 7 follicles per group). (**C**) Representative micrographs of follicles, ovulated COCs and oocytes after 18 h of treatment with hCG for maturation. Oocytes with first polar body extrusion were classified as mature oocytes. Scale bar: 100 μm. (**D**) Real-time qPCR analysis of the follicle development-related genes *Mmp19*, *Zp3* and *Ermp1* in follicles (*n* = 3 per group). (**E**) Real-time qPCR analysis of the oocyte maturation-related genes *Gdf9* and *Bmp15* and the cumulus expansion-related genes *Has2*, *Ptx3*, *Tnfaip6*, *Ptgs2* and *Adamts1* in follicles (*n* = 3 per group). The mRNA expression levels were normalized to the 18 S expression levels. The number of biological replicates is indicated as ‘n’. Data are presented as the means ± SEMs. For (**B**) *P < 0.05; **P < 0.01; ***P < 0.001 versus the *Fshr*^*cre−*^*Stat3*^*f/f*^ + DHEA group. ^##^*P* < 0.01; ^###^*P* < 0.001 versus the *Fshr*^*cre−*^*Stat3*^*f/f*^ + DHEA + IL-22 group

Since STAT3 is known to be a classical downstream signal of IL-22, to explore the role of IL-22 on ovarian function in non-metabolic PCOS, we generated granulosa cell-specific STAT3 knockout (*Fshr*^*cre+*^*Stat3*^*f/f*^) mice. *Fshr*^*cre+*^*Stat3*^*f/f*^ mice were genotyped by PCR analysis, and the knockout efficiency in the *Fshr*^*cre+*^*Stat3*^*f/f*^ mouse granulosa cells was determined by Western blot analyses (Figure S2). By analyzing RNA-seq of granulosa cells from *Fshr*^*cre+*^*Stat3*^*f/f*^ mice, we found that genes related to ovarian follicular development pathway (*Mmp19*, *Zp3*, *Ermp1*) were enriched(Figure S1B), suggesting that STAT3 may play a critical role in follicular development. To further validate our conjecture, first, secondary follicles from *Fshr*^*cre−*^*Stat3*^*f/f*^ mice and *Fshr*^*cre+*^*Stat3*^*f/f*^ mice were separated for in vitro culture. As the results showed, in *Fshr*^*cre−*^*Stat3*^*f/f*^ mice, the follicle diameters slowly increased from 195.12 ± 12.94 μm to 325.01 ± 5.70 μm after DHEA treatment; after supplementation with IL-22, the follicle diameters were significantly higher than those of DHEA-treated follicles on Day 2, Day 4 and Day 6 of culturing (Fig. 3A, B). Moreover, *Mmp19*, *Zp3*, *Ermp1* which were enriched in follicle development pathway by RNA-seq were also reversed by depleting STAT3 (Fig. 3D). Accordingly, the results showed no difference in the follicle diameter between the DHEA and DHEA + IL-22 groups of *Fshr*^*cre+*^*Stat3*^*f/f*^ mice. In addition, after treatment with DHEA and IL-22, the follicle diameters of *Fshr*^*cre+*^*Stat3*^*f/f*^ mice were significantly lower than those of *Fshr*^*cre−*^*Stat3*^*f/f*^ mice. In conclusion, IL-22 failed to improve follicle development when granulosa cell STAT3 was depleted (Fig. 3A, B). In vitro maturation of follicles was performed and yielded the same results. After the depletion of STAT3 in granulosa cells, IL-22 could not promote oocyte ovulation, which was further supported by an unimproved ovulation rate (Fig. 3C) and genes relevant to oocyte maturation and ovulation (Fig. 3E). Taken together, these results suggested that IL-22 can directly improve follicle development and ovulation via STAT3 in PCOS.

### IL-22 directly improves PCOS mice ovarian function dependent on STAT3

**Fig. 4:**
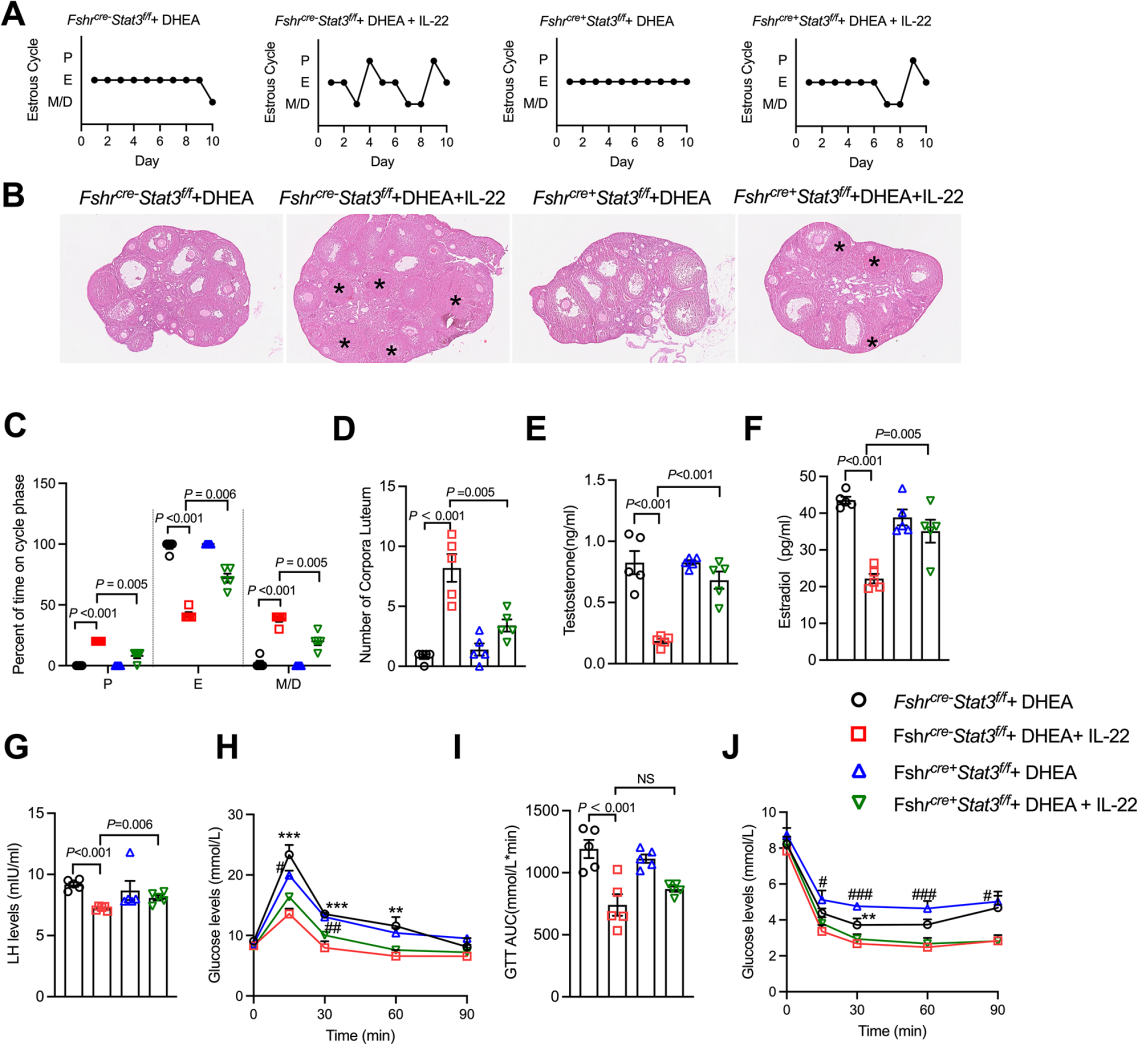
Independent of regulation of metabolism, IL-22 improvement of ovarian function in PCOS is dependent on STAT3. (**A**) Representative estrous cycles of DHEA- and DHEA + IL-22-treated *Fshr*^*cre−*^*Stat3*^*f/f*^ mice and *Fshr*^*cre+*^*Stat3*^*f/f*^ mice. (**B**) Haematoxylin and eosin staining of representative ovaries. The corpora luteum are indicated by asterisks. Images are representative of three independent experiments with similar results. Scale bar: 250 μm. (**C**) Quantitative analysis of estrous cycles (data are presented as the medians with interquartile ranges, n = 5 mice per group). (**D**) Quantitative analysis of corpora luteum (*n* = 5 mice per group). (**E**, **F** and **G**) Serum levels of testosterone, estradiol and LH in DHEA- and DHEA + IL-22-treated *Fshr*^*cre−*^*Stat3*^*f/f*^ mice and *Fshr*^*cre+*^*Stat3*^*f/f*^ mice (*n* = 5 mice per group). (**H**, **I** and **J**) GTT, area under the curve of the GTT and ITT results (*n* = 5 mice per group). The mRNA expression levels were normalized to the 18 S expression levels. The number of biological replicates is indicated as ‘n’. P, proestrus; E, estrous; M/D, metestrus/diestrus. GTT, glucose tolerance test; ITT, insulin tolerance test. Data are presented as the means ± SEMs. For (**H** and **J**) **P < 0.01; ***P < 0.001 versus the *Fshr*^*cre−*^*Stat3*^*f/f*^ + DHEA + IL-22 group. ^#^*P* < 0.05; ^##^*P* < 0.01; ^###^*P* < 0.001 versus the *Fshr*^*cre+*^*Stat3*^*f/f*^ + DHEA + IL-22 group

Next, to further demonstrate that IL-22 depends on STAT3 to directly improve ovarian function, we measured the effects of STAT3 depletion on ovarian function in DHEA-induced PCOS-like mouse models. We found that the administration of IL-22 to DHEA-treated *Fshr*^*cre−*^*Stat3*^*f/f*^ mice significantly alleviated irregular estrous cycle (Fig. 4A, C), polycystic ovaries (Fig. 4B) and increased corpus luteum numbers (Fig. 4D). However this significant improvement was impaired in *Fshr*^*cre+*^*Stat3*^*f/f*^ mice. Compared to those of the DHEA group, serum testosterone, estradiol and LH levels were evidently decreased by IL-22 administration in the presence of STAT3. When granulosa cells were deficient in STAT3, it significantly reversed the ameliorating effect of IL-22 on hormonal abnormalities in DHEA mice (Fig. 4E-G), which further confirmed our speculation that STAT3 plays an important roles in the direct ovarian function improvement of IL-22. Furthermore, the GTT and ITT results showed that insulin resistance in PCOS mice was significantly alleviated by IL-22 (Fig. 4H, J), and the AUC of the GTT results were decreased by IL-22 (Fig. 4I). This suggests that granulosa cell knockdown of STAT3 does not affect the overall amelioration of insulin resistance by IL-22. In conclusion, IL-22 relies on STAT3 to directly mediate the improvement in ovarian dysfunction in PCOS in a metabolically independent manner.

## Discussion

Our study was the first to demonstrate, using a non-metabolic PCOS mouse model, that IL-22 directly improves ovarian function via its classical downstream molecule STAT3, and does not rely on the regulation of systemic metabolic homeostasis. In order to simulate the phenotypic hyperandrogen characteristic of PCOS, DHEA is often used for modeling, but DHEA models have severe metabolic disorders such as insulin resistance. Therefore, we eliminated metabolic symptoms except the ovarian phenotype of PCOS by adding a small dose of rosiglitazone to study the direct effect of IL-22 on the ovarian function of PCOS. To make our proposed pathway even more convincing, we generated granulosa cell-specific STAT3 knockout mice and isolated their ovarian follicles.Our pathway was validated in vitro by superimposing DHEA on cultured mouse follicles to simulate the PCOS ovarian microenvironment according to our previous method [16].

PCOS is highly heterogeneous with different subtypes [18]. Approximately 21-23% of PCOS patients mainly suffer from ovarian dysfunction and do not have metabolic dysfunctions, and it is in these patients that the current therapeutics are limited and nonspecific. In our previous study, IL-22 is previously known to promote browning of white adipose tissue and improve insulin resistance in PCOS [10, 13]. However, all the PCOS models used in previous studies produced severe metabolic disturbances and do not suggest that IL-22 can improve reproductive phenotype in non-metabolic PCOS patients [17]. That means that non-metabolic PCOS models are very difficult to set up, and it is hard to have a suitable method to guarantee that PCOS mice do not have metabolic symptoms. Actually, some studies have reported that the letrozole-induced PCOS model has only a PCOS-like reproductive phenotype without insulin resistance, but the findings do not seem to be consistent: the model does have a contradictory description of metabolic characteristics [19, 20]. Thus, there is an urgent need for a reliable model of non-metabolic PCOS, which will help us to explore the role of IL-22 in the ovarian funtion in PCOS.

As an insulin sensitizer, rosiglitazone was reported to improve some metabolic symptoms in PCOS. Specifically, the use of rosiglitazone significantly improves insulin resistance as well as reduces total cholesterol and triglyceride levels in obese PCOS patients [21]. Ovarian-only phenotype in patients with non-metabolic PCOS, and due to androgens are mainly derived from ovarian theca cells, the level of androgens reflects the ovarian function in non-metabolic PCOS to a certain extent. Thus, if the effect of rosiglitazone on ovarian function is observed using androgen levels as an indicator, the role of rosiglitazone in improving ovarian function in PCOS patients remains controversial. Levalle et al. applied a regimen of 4 mg rosiglitazone daily for 3 months in recruited PCOS patients, and they found that androgens remained unchanged, but the level of LH was decreased [22]. Accordingly, Reaven et al. reproduced the fact that androgens were not changed by using 8 mg of rosiglitazone daily for 3 months, but LH did not decrease in their clinical trials [23]. In comparison, Huang et al. found that rosiglitazone 4 mg daily for 6 months significantly reduced testosterone levels [21]. Therefore, we hypothesize that the improvement in ovarian function in PCOS patients relies heavily on the dose and duration of rosiglitazone administration, specifically that short-term use of rosiglitazone may preferentially improve insulin resistance without restoring ovarian function in PCOS patients. Consistantly, our data showed that a low dose of rosiglitazone (3 mg/kg/d, normally 8-10 mg/kg/d) ameliorated metabolic dysfunction in the DHEA-induced PCOS mouse model and reversed the irregular estrous cycle to some extent, whereas rosiglitazone had no impact on sex hormone levels. After rosiglitazone treatment, the main phenotypes of the DHEA-induced PCOS mouse model were PCOM, irregular estrous cycle, and hyperandrogenism, which basically simulated the clinical features of non-metabolic PCOS. Overall, DHEA + rosiglitazone may well mimic a non-metabolic PCOS mouse model.

PCOS is usually categorized into 4 subtypes (A/B/C/D) based on the Rotterdam criteria. Phenotype A includes androgen excess, oligo-anovulation and PCOM, Phenotypes B and C are defined by androgen excess, oligo-anovulation and androgen excess, PCOM respectively. Only oligo-anovulation and PCOM fall into phenotype D. However, recent studies have proposed a new interpretation of the Rotterdam criteria: since both A/B/C phenotypes are characterised by hyperandrogenism, phenotype D is regarded as the only subtype that explains ovarian pathogenesis [24, 25]. The fact that A/B/C both exhibit considerable metabolic disorders and exacerbate reproductive symptoms makes it difficult to distinguish the underlying pathogenesis of PCOS. In particular, it seems that phenotype D PCOS patients, which characterized by oligo- or anovlation and PCOM, were mainly caused by problems with the ovaries themselves. However, current animal models for PCOS do not reflect the characteristics of phenotype D. Therefore, different animal models for different PCOS phenotypes are needed to better investigate therapeutic approaches. In our non-metabolic PCOS mouse model, rosiglitazone eliminates insulin resistance in DHEA mice but preserves the disordered estrous cycle, PCOM, and testosterone abnormalities in PCOS mice. This animal model is based on the typology described by Myers et al. and more closely resembles PCOS (formally known as phenotype D PCOS) [26], and although still hyperandrogenic, it is still an important animal model for the study of phenotype D PCOS.

We then used this model to explore the possibility of IL-22 as an etiologic treatment for PCOS patients without metabolic abnormalities and the potential mechanisms behind it. Our result shows that the administration of IL-22 evidently improved ovarian function and alleviated hyperandrogenism in this non-metabolic PCOS mouse model, indicating that IL-22 could directly target PCOS ovaries to modulate the process of follicle development and ovulation.

Considering that STAT3 is a classical downstream molecule of IL-22 [27], we generated granulosa cell-specific STAT3 knockout mice. Due to the absence of metabolic abnormalities PCOS patients are predominantly characterized by significant reproductive symptoms, such as impaired follicular development and ovulatory dysfunction [28]. We applied DHEA in an in vitro follicular culture system to simulate PCOS follicular developmental block and ovulation impairment, we visualized that IL-22 treated follicles grew better and ovulated at a higher rate than the DHEA group and IL-22 restored the expression of follicular development marker genes (*Mmp19*, *Zp3*, *Ermp1*) and ovulation marker genes (*Gdf9*, *Bmp15*, *Ptx3*, *Tnfaip6*, *Adamts1*, *Ptgs2*), which was not the case in the *Fshr*^*cre+*^*Stat3*^*f/f*^ mice. Moreover, we observed that after DHEA treatment, *Fshr*^*cre+*^*Stat3*^*f/f*^ mice were characterized by an irregular estrous cycle, PCOM and elevated serum androgen levels, and these abnormalities were not significantly relieved by IL-22 compared with *Fshr*^*cre−*^*Stat3*^*f/f*^ mice. Indeed, the *Fshr*^*cre+*^*Stat3*^*f/f*^ mice themselves had some degree of impaired follicular development and ovulation, with most of their follicular development, oocyte maturation and cumulus expansion related genes downregulated(*Gdf9*, *Bmp15*, *Ptx3*, *Adamts1*, *Mmp19*, *Zp3*, *Ermp1*). However, in our in vitro culture data of *Fshr*^*cre+*^*Stat3*^*f/f*^ mice follicles, impaired follicular growth was shown only on day 6, which may imply that STAT3 plays a greater role in preovulatory follicles. Thus the role of STAT3 in different stages of follicle development still deserves to be explored in depth.

Overall, the combination of this phenomenon again suggests that IL-22 can directly improve ovarian follicular development and ovulation through its classical downstream STAT3 to alleviate PCOS model. In conjunction with our previous findings, which suggests that PCOS patients with reduced serum IL-22 levels, Together, these studies suggest that IL-22 may be a future treatment for non-metabolic PCOS. However the limitation of our study is that it is still validated in animal models, and since IL-22 is an inflammatory factor with a wide range of actions throughout the body, which may not play the same role in different organs and models. In addition, there is no conclusive evidence on the dosage and safety of IL-22 [11]. Thus our study provides further support for the exploration of IL-22 for the treatment of non-metabolic PCOS.

## Conclusion

In our study, we constructed a non-metabolic PCOS mouse model and report the specific mechanism by which IL-22 directly improves ovarian function independently of regulating metabolism: IL-22 improves follicular development and ovulation in PCOS mice via STAT3. This study reveals the possibility of IL-22 for the treatment of non-metabolic PCOS, but more clinical studies are still required.

### Electronic supplementary material

Below is the link to the electronic supplementary material.

Supplementary Material 1

## Data Availability

No datasets were generated or analysed during the current study.
