# Supplementary material for: Interlukin-22 improves ovarian function in polycystic ovary syndrome independent of metabolic regulation: a mouse-based experimental study
Source: J Ovarian Res. 2024 May 11;17:100. doi: 10.1186/s13048-024-01428-x (PMC11088773; doi:10.1186/s13048-024-01428-x)
Supplement: Supplementary file 1 — Supplementary Material 1 [file 13048_2024_1428_MOESM1_ESM.docx]

Table S1. Primer sequences used in quantitative real-time PCR analysis and genotype.

| Target genes | Primer sequence |
| --- | --- |
| *18S* (mouse) | Forward 5’- GAAACGGCTACCACATCCAAGG -3’ |
|  | Reverse 5’- GCCCTCCAATGGATCCTCGTTA -3’ |
| *Gdf9* (mouse) | Forward 5’-TCTTAGTAGCCTTAGCTCTCAGG -3’ |
|  | Reverse 5’-TGTCAGTCCCATCTACAGGCA -3’ |
| *Bmp15* (mouse) | Forward 5’-TCCTTGCTGACGACCCTACAT -3’ |
|  | Reverse 5’-TACCTCAGGGGATAGCCTTGG -3’ |
| *Has2* (mouse) | Forward 5’-TGTGAGAGGTTTCTATGTGTCCT -3’ |
|  | Reverse 5’-ACCGTACAGTCCAAATGAGAAGT -3’ |
| *Ptx3* (mouse) | Forward 5’-CCTGCGATCCTGCTTTGTG -3’ |
|  | Reverse 5’-GGTGGGATGAAGTCCATTGTC -3’ |
| *Adamts1* (mouse) | Forward 5’-CATAACAATGCTGCTATGTGCG -3’ |
|  | Reverse 5’-TGTCCGGCTGCAACTTCAG -3’ |
| *Tnfaip6* (mouse) | Forward 5’-GGGATTCAAGAACGGGATCTTT -3’ |
|  | Reverse 5’-TCAAATTCACATACGGCCTTGG -3’ |
| *Mmp19*（mouse） | forward 5′- CTGTGGCTGGCATTCTTACTT-3′ |
|  | reverse 5′- GGGCAGTCCAGATGCTTCC-3′ |
| *Zp3* (mouse) | Forward 5’- ATGGCGTCAAGCTATTTCCTC-3’ |
|  | Reverse 5’- CGTGCCAAAAAGGTCTCTACT-3’ |
| *Ermp1* (mouse) | Forward 5’- CTCGCACTCTACCTGCTCG-3’ |
|  | Reverse 5’- CCAATGGCCGTTATGTGTTCC-3’ |
| *Fshr* （mouse） | Forward 5’- CTCTGGTGTAGCTGATGATC-3’ |
|  | Reverse 5’- TAATCGCCATCTTCCAGCAG-3’ |
| *Stat3* （mouse） | Forward 5’- CTCTGGTGTAGCTGATGATC-3’ |
|  | Reverse 5’- TAATCGCCATCTTCCAGCAG-3’ |

***
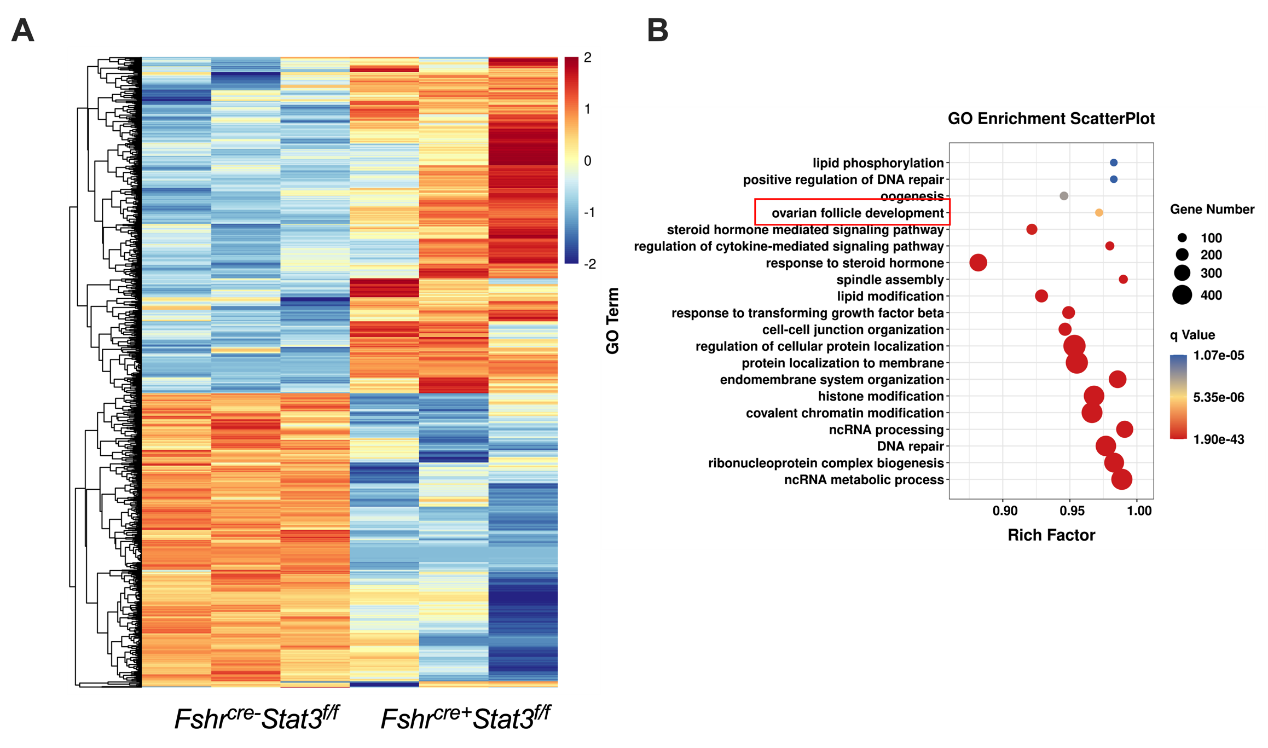
***

**Figure S1. RNA-seq of *Fshr^cre+^Stat3^f/f^* mice granulosa cells.**

(A) Heatmap of the differentially expressed genes between granulosa cells collected from *Fshr^cre-^Stat3^f/f^* mice and *Fshr^cre+^Stat3^f/f^* mice. (B) Pathways significantly enriched by Gene Ontology analysis.


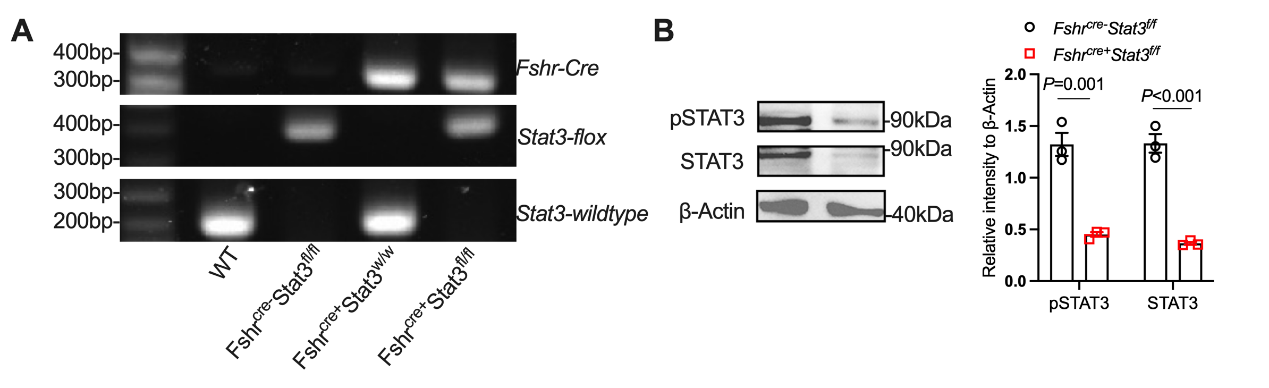


**Figure S2. STAT3 was selectively depleted in the granulosa cells of *Fshr^cre+^Stat3^f/f^* mice.** (A) Genotype results analysed by PCR. (B) Western blot analysis of pSTAT3 and total STAT3 in granulosa cells of *Fshr^cre-^Stat3^f/f^* and *Fshr^cre+^Stat3^f/f^* mice. β-Actin was loading control.
